# Effort and substance use: differentiating tobacco use through reinforcement learning of effort based decision making

**DOI:** 10.1007/s10827-026-00953-6

**Published:** 2026-08-28

**Authors:** Kasey P. Spry, Jazmyne James, Alison H. Oliveto, Michael Mancino, Kenneth T. Kishida, Merideth A. Addicott

**Affiliations:** 1 Department of Translational Neuroscience, Wake Forest University School of Medicine, Winston-Salem, NC USA; 2 Neuroscience Graduate Program, Wake Forest University Graduate School of Arts and Sciences, Winston-Salem, NC USA; 3 Department of Psychiatry, University of Arkansas for Medical Sciences, Little Rock, AR USA; 4 Department of Biology, Wake Forest University, Winston-Salem, NC USA; 5 Department of Neurosurgery, Wake Forest University School of Medicine, Winston-Salem, NC USA

**Keywords:** Effort, Tobacco, Opioids, Reinforcement learning, Smoking, Decision-making

## Abstract

Effort-based decision making evaluates rewards relative to the effort required to obtain it, an important process of healthy goal-directed motivation and behavior. Computational models provide mechanistic insights underlying choice behavior and potential alterations in neuropsychiatric disorders, including substance use disorders. We applied computational models to effort-based choice behavior to characterize underlying decision processes and if these mechanisms differ by substance use status. Participants completed the Effort Expenditure for Rewards Task, choosing between low- and high-effort options for monetary rewards varying in magnitude and probability. Participants met criteria for no tobacco use (n = 23), current tobacco use disorder (n = 26), former tobacco use disorder (n = 22), and tobacco and opioid use disorder (n = 29). Computational models from two families, Subjective Value and Reinforcement Learning, were fit and compared. Parameters from the best-fitting model underwent principal components analysis and linear discriminant analysis. Temporal difference reinforcement learning model demonstrated greater model evidence and predictive accuracy, indicating better fit to effort-based choice behavior. Principal components analysis revealed meaningful multivariate distinctions: PC1 differentiated all groups except individuals without tobacco use versus individuals with current tobacco use disorder; PC3 distinguished tobacco and opioid use disorder from all other groups. Linear discriminant analysis demonstrated group separation with 84% classification accuracy. A reinforcement learning framework better explained participants’ effort-based choice behavior. Substance use status relates to dynamic behavioral changes (i.e. learning) as measured by the multivariate combination of learning rate, future discounting, and choice temperature.

**Supplementary Information:**

The online version contains supplementary material available at https://doi.org/10.1007/s10827-026-00953-6.

## Introduction

An important feature of healthy decision-making is the willingness to exert effort, either physical or cognitive effort, to obtain rewards. Effort-based decision making (EBDM) evaluates the value (i.e. magnitude and probability) of a reward in relation to the effort required to obtain it (Salamone et al., 2016). If effort expenditure is perceived as increasingly costly, the reward may become devalued (Inzlicht et al., 2018; Klein-Flügge et al., 2015). These cost-benefit analyses are an important process of goal-directed motivation and behavior, which allows individuals to obtain goals despite obstacles in the environment (Bailey et al., 2016). Impairments in EBDM have been linked to reduced motivation and dysfunction in several psychiatric conditions, including substance use disorders (SUD).

Traditionally goal-directed behaviors are analyzed with generalized estimating equations or other repeated measures analyses to understand *what* choices are made (e.g. proportion of high/low effort option selections) and how this differs by diagnosis or group classification. There has been an increased focus on developing mechanistic accounts of the computational processes underlying goal-directed motivation and behavior, specifically EBDM (Huys et al., 2016). For example, computational models have been applied to EBDM tasks to investigate *how* choices are made (e.g. with or without systematic utilization of given information, learning from previous outcomes, etc.) (Cooper et al., 2019; Jarvis et al., 2022; Kuhn et al., 2025; Whitton et al., 2024). Cooper et al. (2019) used a computational approach on an EBDM task, the Effort Expenditure for Rewards Task (EEfRT), using models that vary in terms of their utilization of trial-wise information (reward magnitude, reward probability, and effort allocation) to guide choice behavior. While these types of models offer insight into how individuals use reward and effort information, they might not capture key details such as the influence of previous choices or outcomes (i.e., learning). This is an important limitation of these models because previous choices and outcomes influence succeeding choice behavior via reinforcement learning mechanisms (O’Doherty et al., 2003; Schultz et al., 1997; Sutton & Barto, 1998; Watkins & Dayan, 1992).

Reinforcement learning (RL) models are defined by the ability to learn from interactions with an environment through trial and error to maximize cumulative reward outcomes. One class of RL models, Rescorla-Wagner RL models (defined by their ability to model learning as changes in associative strength between cues and outcomes), were applied to an EBDM paradigm that combined a probabilistic reward task with physical effort. This model showed that effort results in more efficient learning from positive outcomes and less efficient learning from negative outcomes. This effect varies across individuals and is more pronounced in those who are more averse to effort at baseline (Jarvis et al., 2022).

A related and widely used class of RL models are temporal difference reinforcement learning (TDRL) models, defined by their ability to learn predictions of future rewards over time by updating value estimates based on differences between successive predictions (i.e., prediction errors). Importantly, TDRL models provide a neurocomputational account of decision-making that closely aligns with dopaminergic signaling, as phasic activity of midbrain dopamine (DA) neurons has been shown to encode prediction errors (Hart et al., 2014; Liebenow et al., 2022; Montague et al., 1996; Sands et al., 2023a, b; Schultz et al., 1997; Sutton and Barto, 1998). Consistent with this framework, extensive research with animal and human paradigms indicates DA modulation of EBDM, indicating that increasing or decreasing DA transmission enhances or diminishes (respectively) the willingness to expend effort for rewards (Assadi et al., 2009; Caul & Brindle, 2001; de Jong et al., 2015; Floresco et al., 2008; Kurniawan, 2011; Salamone et al., 2007, 2016, 2018, 2022; Wardle et al., 2011). Mesolimbic DA is thought to regulate the activational component of motivation, specifically the willingness to exert effort for rewards, rather than only reward valuation. These findings position dopaminergic systems as a key mechanistic link between reward processing and effort expenditure.

Despite this strong theoretical link, TDRL models have not, to our knowledge, been applied to EBDM paradigms. Thus, TDRL models may help clarify processes underlying decision-making behavior modulated by changes in DA signals, including EBDM.

Individual and group differences in RL and DA signaling are particularly relevant when considering populations characterized by altered reward processing, such as individuals with SUD. Chronic use of substances such as nicotine and opioids has been associated with dysregulation of the mesolimbic DA system, which is critical for encoding prediction errors and RL (Ashok et al., 2019; Branco et al., 2025; Dagher et al., 2001; Fehr et al., 2008; Volkow et al., 2019; Wills et al., 2022; Wittenberg et al., 2020). This dysregulation may influence how effort costs and reward benefits are integrated over time, potentially leading to differences in subjective value and learning dynamics captured by TDRL. A small but growing number of studies have investigated the effects of SUDs on EBDM. One study reported that individuals with tobacco use disorder (TUD) had diminished sensitivity to nondrug reward values (Addicott et al., 2020), while other studies have reported no effects of smoking status, acute nicotine, or tobacco withdrawal on physical EBDM (Mitchell, 2004; Mitchell, Suzanne H., 1999; Nunez et al., 2022). However, these studies did not apply RL models to EBDM behavior. Examining EBDM through a RL framework in populations with substance use provides an opportunity to link observed choice behavior with underlying neurocomputational mechanisms, rather than solely descriptive group-level differences in choice behavior.

To this end, we investigated effort-based choice behavior with subjective value and RL models to better understand *how* individuals are making effort-based choices and if these mechanisms differ by group classification, here substance use status. In this report, we are using data published previously comparing EEfRT performance among participants with current, former, and never tobacco use (Addicott et al., 2020). These data were previously analyzed using generalized estimating equations. The analysis of computationally modeled EEfRT choice behavior in these groups is not simply a reanalysis of existing work (i.e. sensitivity of groups to given information) but instead offers insights into group behavior in the use of given information to drive choice behavior between the two options. In essence, our focus here is to determine *how* individuals made EBDM choices as opposed to *what* EBDM choices were made. We also expanded this dataset to include individuals with both OUD and TUD who completed the EEfRT as part of a different study. Over 80% of individuals with opioid use disorder (OUD) use tobacco; however, dual use is rarely studied (John et al., 2019) and the specific effects of tobacco use on behavior need to be controlled when investigating individuals with OUD (iOUD). Furthermore, if EBDM is influenced by substance use-related changes, then there may be synergistic effects of dual opioid and tobacco use in EBDM behavior.

We hypothesize that TDRL will be a better explanation of behavior on the EEfRT and will better predict unobserved EEfRT choice behavior data. This would be consistent with the established role of dopaminergic signaling in EBDM and RL, as well as known alterations in DA in TUD and OUD. Secondarily, we hypothesize that substance use status will differ in the multivariate combination of TDRL posterior parameters. This would be consistent with distinct patterns of reward processing and decision-making associated with different substance use states with differences in current use, history of use, and co-occurring substance use, each of which may differentially influence dopaminergic signaling and RL processes. As such, we expect that these groups will exhibit distinct computational pattens of learning and choice behavior.

## Methods

### Participants

#### Study 1 (Addicott et al., 2020)

Participants were recruited from Durham, NC USA (*n* = 28) and Little Rock, AR USA (*n* = 43) between August 2016 and October 2018 and met criteria for current TUD (*n* = 26, Current TUD), former TUD (*n* = 22, Former TUD), and no tobacco use (*n* = 23, Never TUD). Participants were 18–55 years old. Current TUD smoked ≥ 10 cigarettes/day for ≥ 2 years, expired breath carbon monoxide (CO) concentration of ≥ 10 ppm, morning urinary cotinine concentration of > 100 ng/ml, and no regular use of electronic cigarettes/other nicotine/tobacco products. Former TUD smoked ≥ 10 cigarettes/day for ≥ 2 years, reported no tobacco use ≥ 12 months, breath CO ≤ 5 ppm, and urinary cotinine < 100 ng/ml. Never TUD smoked < 50 cigarettes in their lifetime, reported no use of tobacco or nicotine ≥ 6 months, breath CO ≤ 5 ppm, and urinary cotinine < 100 ng/ml. Participants were screened using structured interviews for medical, psychiatric, and drug use history. Exclusion criteria included serious health problems (e.g. liver, lung or heart disease), severe psychiatric disorders that interfere with day-to-day function, any history of psychotic disorders, use of anti-psychotics or second-line pharmacotherapies for depression/anxiety, a history of neurologic disorders, serious head trauma, or drug/alcohol dependence in the past 6 months (other than tobacco). Participants were also excluded if they tested positive for drugs, alcohol, or pregnancy. Participants provided written informed consent, and this protocol was approved by Duke University’s and University of Arkansas for Medical Sciences’ Institutional Review Boards. Computational analysis was conducted at Wake Forest University School of Medicine.

#### Study 2

Participants enrolled in R01 DA039088 (MPI: Oliveto & Mancino; See (Ray et al., 2025) between April 2018 and February 2021 at the University of Arkansas for Medical Sciences in Little Rock, AR USA completed the EEfRT. Participants were 18–65 years old, sought treatment for opioid use, fulfilled DSM-5 criteria for moderate to severe OUD, and provided a urine sample negative for benzodiazepines and barbiturates. Of the 44 participants who completed the EEfRT, *n* = 29 smoked > 5 cigarettes/day and were included in this analysis (TUD + OUD). Exclusion criteria included unstable medical conditions or stable conditions that would interact with study procedures, seizure disorder, severe psychiatric disorders requiring psychoactive medication, taking prescription drugs that would interact with the study drugs, and physical dependence on drugs other than opioids, tobacco, or cannabis. See Ray et al., (2025) for complete details. Participants who used alternative forms of nicotine/tobacco (*n* = 7), those who did not use nicotine/tobacco (*n* = 5), and those who did not use > 5 cigarettes/day (*n* = 2) were excluded from the analysis due to their small sample sizes. Participants provided written informed consent, and this protocol was approved by University of Arkansas for Medical Sciences’ Institutional Review Boards. Computational analysis was conducted at Wake Forest University School of Medicine.

### Procedure

This analysis combines data from two studies that used identical versions of the EEfRT. Study 1 included Current TUD, Former TUD, and Never TUD while Study 2 included TUD + OUD. While the study procedures were similar, they are described separately below.

#### Study 1

Individuals with TUD (iTUD) were asked to smoke as usual prior completing a 3-h study visit where the EEfRT was administered at the end of the visit. As part of the EEfRT training, study staff read through the instructions with the participants, and then participants completed four practice trials consisting of at least one low-effort and one high-effort task. After addressing any questions, participants completed the EEfRT. Immediately prior to the EEfRT, participants completed the Shiffman-Jarvik Withdrawal Scale (Shiffman & Jarvik, 1976) to assess six common symptoms of nicotine withdrawal: craving, negative affect, arousal, somatic symptoms, appetite, and habit withdrawal. Never TUD participants completed non-craving/habit withdrawal questions to assess general/nonspecific discomfort.

#### Study 2

This study examined adjunct gabapentin during outpatient buprenorphine detoxification to improve OUD treatment outcomes. All data analyzed were collected prior to buprenorphine dosing, with the exception of one participant. At the first study visit, after completing eligibility requirements, participants provided a urine sample and completed assessments including the Subjective Opiate Withdrawal Scale (SOWS), consisting of 16 items describing possible opioid withdrawal symptoms they might be experiencing at the time of survey completion rated on a scale from 0 (not at all) to 4 (extremely) (Handelsman et al., 1987). Then participants completed the EEfRT with instructions read by study staff and completing four practice trials consisting of at least one low-effort and one high-effort task.

### Effort Expenditure for Rewards Task (Treadway et al., 2009)

**Fig. 1:**
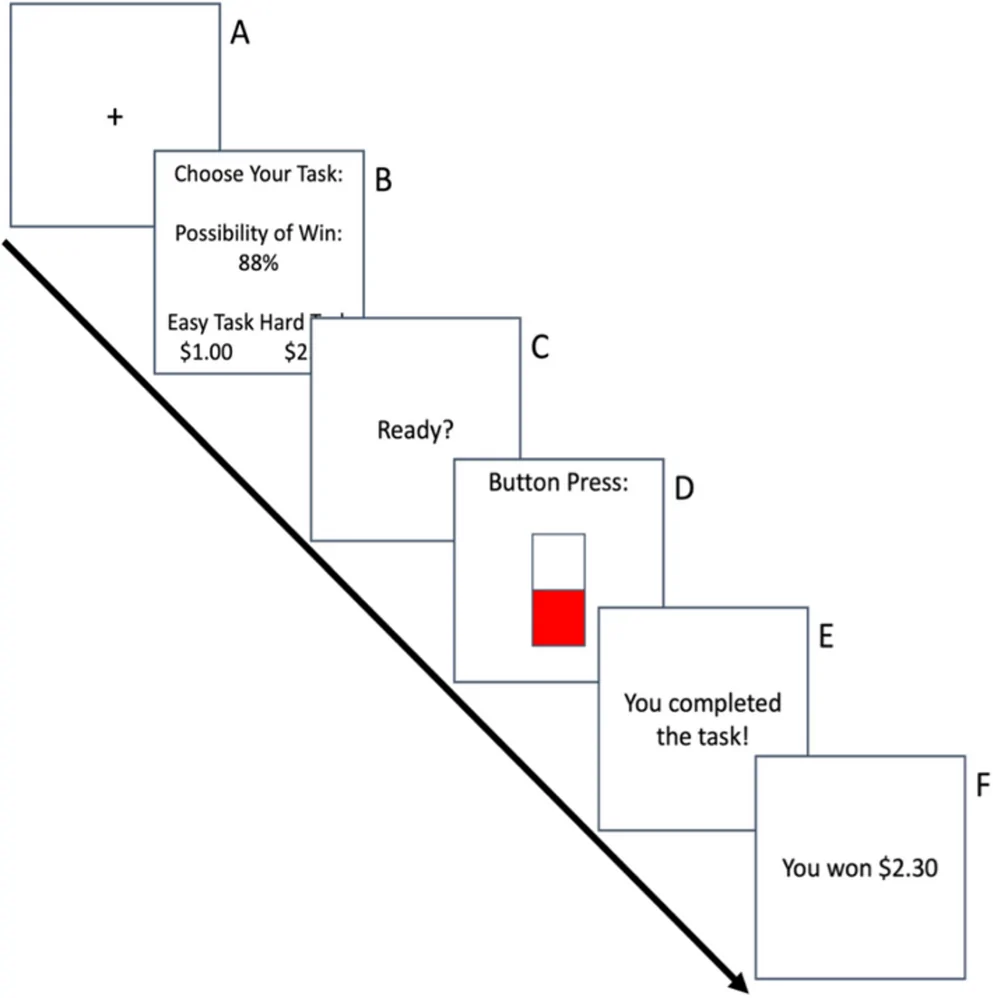
**Effort Expenditure for Rewards Task**. Depiction of a single EEfRT trial. (**A**) 1 s fixation cue. (**B**) Five sec choice period in which participants are presented with the reward magnitude and probability. (**C**) 1 s “Ready” screen. (**D**) Action screen where participants make rapid button presses. Easy Task: 30 button presses using the dominant index finger in 7 s. Hard Task: 100 button presses using the non-dominant pinky finger in 21 s. (**E**) 2 s Feedback on completion/incompletion. (**F**) 2 s Monetary reward feedback

The EEfRT is a multi-trial, two-choice, cost-benefit task designed to measure one’s willingness to exert effort (Treadway et al., 2009). Figure 1 depicts a single EEfRT trial. Participants are given the opportunity to choose between two different task difficulty levels, hard and easy, to obtain monetary rewards. Both task options consist of repeated manual button presses within a timeframe. Complete button presses are represented onscreen by the height of a vertical bar. The low-effort, easy task option requires 30 button presses with the dominant index finger within 7 s. The high-effort, hard task option requires 100 button presses with the nondominant little finger within 21 s. Participants are monitored during the task to ensure that they use the correct finger for each task.

In low-effort trials, participants can receive $1.00 if they complete the task on time. In high-effort trials, participants can receive a variable reward between $1.24 and $4.30 (i.e. reward magnitude). Across trials (both low- and high-effort tasks), the likelihood of receiving a reward upon successful completion of the trial is either 12%, 50%, or 88% (i.e. reward probability). At the start of each trial, participants are shown the reward magnitude for both task options and the probability level. Participants have 5 s to make a choice; if a task is not selected, they are randomly assigned to a task. After selecting the task, they complete the button press task and immediately receive feedback informing them if the task is completed successfully and whether they received the reward for that trial. Participants are informed that a single trial that resulted in a reward will be selected at random at the end of the EEfRT and the participant would be given this amount (between $1 and $5) as bonus pay.

Low-effort trials last approximately 15 s and high-effort trials last approximately 30 s. Participants are told they have 20 min to play as many trials as possible. They are informed of the trade-off between choosing too many high-effort trials early in the game then missing out on playing large reward magnitude, large probability trials later in the game to discourage the exclusive selection of either the low-effort or high-effort task. Since participants could complete a variable number of trials, only data from the first 50 trials was used for consistency, consistent with previous EEfRT studies (Treadway et al., 2012).

The EEfRT was programmed in MATLAB (Mathworks Inc., Natick, MA) using the Psychtoolbox version 3.0.

### Data analysis

Prior work examined EEfRT choice behavior in Current, Former, and Never TUD using generalized estimating equation (Addicott et al., 2020). The current secondary analysis extends this work by computationally modeling the dynamics of EEfRT choice behavior in these groups and adding the TUD + OUD cohort to offer insights into group behavior in the use of given information and learning effects in driving choice behavior between the two options.

Participant demographics were analyzed using one-way analysis of variance (ANOVA) with Bonferroni post hoc comparisons for means and chi-square tests for categorical counts. EEfRT performance was analyzed using one-way ANOVA.

To formally test how effort, reward information (magnitude and probability), and feedback drive effort-based choices, we compared two families of computational models, subjective value models and RL models.

### Subjective value computational models (Cooper et al., 2019)

#### Full subjective value model

The ‘full subjective value model’ (Full SV model, Eq. 1) will hypothetically fit best for participants who consistently incorporate trial-wise reward and probability information when allocating effort (Cooper et al., 2019). The subjective value of a given trial is calculated by taking the objective reward, R ($1.00 - $4.30), scaling it by the reward probability, P (0.12, 0.50, 0.88), and reducing it by the amount of effort required to obtain it (0.3 for the low-effort trials or 1.0 for the high-effort trials).

1 $$\begin{array}{c}SV=R*\:{P}^{h}-kE\end{array}$$

Free parameters, *k* and *h*, capture the individual differences in which reward is discounted by effort and probability of receiving the reward by weighing the components. The 0 < *k <* 10 parameter influences subjective value based on the amount of effort required, thus effort that is perceived as costly is reflected by a higher value of *k*. The 0 < *h <* 10 parameter influences subjective value by weighting the probability of receiving the reward.

#### Reward only subjective value model

Some participants may allocate effort based on reward magnitude alone (Cooper et al., 2019). The reward only subjective value model (Reward Only SV model, Eq. 2) will hypothetically fit best for participants who consistently incorporate trial-wise reward magnitude information but not reward probability information when allocating effort. The subjective value of a given trial is calculated by taking the objective reward, R ($1.00 - $4.30), and reducing it by the amount of effort required to obtain it (0.3 for the low-effort trials or 1.0 for the high-effort trials).

2 $$\begin{array}{c}SV=R-kE\end{array}$$

Free parameter, *k*, captures the individual differences in which reward is discounted by effort. The 0 < *k <* 10 parameter influences subjective value based on the amount of effort required, thus effort that is perceived as costly is reflected by a higher value of *k*.

#### Softmax choice policy function

Subjective values (SVs) estimated in the Full SV and Reward Only models are used to estimate choice behavior according to a ‘policy’ function. The policy function defines the strategy for selecting an option based on the current state. These SVs are transformed into probabilities (Eq. 3) of selecting the low-effort or high-effort option using the Softmax choice policy (Sutton & Barto, 1998). This policy function calculates choice probabilities, where the probability, * P*, of choosing a given option, * choice*, on each trial, * t*, was estimated based on its subjective value relative to that of the alternate option. Free parameter 0 < *β* < 100 is an inverse choice temperature parameter that captures individual differences in choice stochasticity where it determines the Softmax function slope. Lower inverse temperature values lead to a more randomized choice selection policy and higher inverse temperature values lead to a more exploitative and greedy choice selection policy.

3 $$\begin{array}{c}P\left({choice}_{t}=opt1\:|\:{SV}_{opt1}\:,\:{SV}_{opt2}\right)=\:\frac{{e}^{{SV}_{opt1}*\beta\:}}{{e}^{{SV}_{opt1}*\beta\:}+\:{e}^{{SV}_{opt2}*\beta\:}}\end{array}$$

### Reinforcement learning computational model (Sutton, 1988; Sutton & Barto, 1998; Watkins & Dayan, 1992)

#### Temporal difference reinforcement learning model

In model-free temporal difference reinforcement learning (TDRL), agents learn from temporal difference reward prediction errors (TD-RPE, $\:{\delta\:}_{i}$, Eq. 4).

4 $$\begin{array}{c}{\delta\:}_{i}=\:\left[{outcome}_{i}+\gamma\:\underset{a}{\mathrm{max}}\:Q\left({s}_{i+1},\stackrel{\sim}{a}\right)\right]-Q\left({s}_{i},{a}_{i}\right)\end{array}$$

Here, TD-RPEs are the error between the predicted and actual experiences where $\:{outcome}_{i}$ is the actual outcome (positive or negative) experienced in state $\:{s}_{i}$ after taking action $\:{a}_{i}$, 0 < γ < 1 is a temporal discount parameter that discounts future expected value, and $\:\underset{a}{\mathrm{max}}\:Q({s}_{i+1},\stackrel{\sim}{a})\:$is the maximum expected value over all actions $\:\stackrel{\sim}{a}$ afforded in the next state $\:{s}_{i+1}$.

Following an action-contingent reward or punishment (i.e., $\:{outcome}_{i}$), the TD-RPE provides an update value for the current state-action pair, $\:Q({s}_{i},{a}_{i})$, according to a ‘Q-learning’ rule (Eq. 5).

5 $$\begin{array}{c}Q\left({s}_{i},{a}_{i}\right)\leftarrow\:Q\left({s}_{i},{a}_{i}\right)+\alpha\:{\delta}_{i}\end{array}$$

Where * i* indicates discrete time points within a trial and 0 < α < 1 is the learning rate that parameterizes how much the agent weighs a TD-RPE, $\:{\delta\:}_{i}$, during learning. In the context of the EEfRT, we defined the discrete time points within a trial as * i* = {1,2,3} [1, options presented; 2, action taken; 3, outcome and reward presented]. Also, within the context of the EEfRT, $\:Q({s}_{i},{a}_{i})$ represents the expected value associated with selecting a given option on the ‘Option Presentation’ screen (Fig. 1B) and represents that action’s expected future reward.

#### Softmax choice policy function

As above, we modeled participant choice behavior according to the Softmax choice policy (Sutton & Barto, 1998). This policy function calculates choice probabilities (Eq. 3), whereby the probability, * P*, of choosing a given option, * choice*, on each trial, * t*, was estimated based on its learned Q-value, $\:Q\left({s}_{i},{a}_{i}\right)$, relative to that of the alternate option.

### Model fitting

We fit each model using hierarchical Bayesian analysis. Individual level parameter values are drawn from group level distributions over each model parameter. This approach derives individual participant parameter values as deviations from a group mean. For a more detailed description of the hierarchical Bayesian procedure, see (Sands et al., 2023a, b). On trials where participants did not make a choice within 5 s, they were randomly assigned to a task. Non-self-initiated trials accounted for 39 out of 5000 trials (0.78%) across all participants. In the computational analysis, randomly assigned trials were included for learning updates, because participants still performed and observed task outcomes that could plausibly contribute to learning irrespective of whether the choice was self-initiated. These randomly assigned trials were excluded from predictive evaluation because the observed choice does not reflect participants’ decision policy and including such trials in predictive metrics would artificially penalize models by evaluating them against stochastic, imposed choices rather than participant-generated decisions. This approach ensures that learning dynamics reflect all experienced outcomes while preserving the interpretability and fairness of model comparison based on voluntary choice behavior.

All models were fit in Stan using the Hamiltonian Monte Carlo (HMC) sampling algorithm via the R package rstan (version 2.32.6) to estimate the joint posterior distributions for the Subjective Value Models and RL Model across all cohorts simultaneously, with individual parameters hierarchically nested within cohort-level distributions. For all models, we ran four Markov chains, with 10,000 total samples per chain (2,000 warm-up) for a total of 32,000 posterior samples per model parameter. To confirm proper model fitting, we inspected the convergence of the Markov chains by verifying the Gelman-Rubin $\:\widehat{R}$ (i.e., the ratio of within- and between-chain variance), which was approximately or less than 1.1 for almost all parameters across all models.

### Model comparison

We compared the fit of each model to participant choice behavior on the EEfRT according to model evidence estimates (i.e. Bayesian marginal likelihood), which represents the probability or plausibility of observing the actual EEfRT data under each model (MacKay, 2003). The marginal likelihood for each model is an optimal measure for performing model comparison as it represents the balance between the fit of each model to the data and the complexity of each model. In Bayesian model comparison, the model with the greatest model evidence (i.e. least negative) is deemed the best explanation for the data. We estimated the marginal likelihood for all models using bridge sampling as implemented in the R package bridgesampling [v. 1.1-2; (Gronau et al., 2020)]. Bridge sampling is a statistical technique used to estimate marginal likelihoods of models even with hierarchical structure. We performed 10 repetitions of the sampler and report the median and interquartile range of the estimates of model evidence.

As a secondary comparison, we estimated each model’s Bayesian leave-one-out (LOO) cross-validation predictive accuracy, defined as a model’s expected log predictive density [ELPD-LOO; (Vehtari et al., 2017)]. The ELPD is a statistical technique used to assess how well a statistical model predicts new data by providing an estimate of the predictive accuracy of the model. It focuses on how well the model generalizes to unseen data while penalizing overfitting to the data and it compares models by their predictive performance, helping select the model that balances complexity and fit. The model with the greatest ELPD (i.e. least negative) is deemed the best explanation for the data. We estimated the ELPD for all models for each cohort separately using the R package loo [v. 2.8; (Vehtari et al., 2024)].

### Unsupervised dimensionality reduction and group classification

We performed a principal components analysis (PCA) to reduce dimensionality of resulting parameter sets and assess whether distinct covariation amongst the three-vector of TDRL posterior parameter estimates could distinguish between groups. The maximum a posteriori estimate (MAP; the parameter value that maximizes the posterior distribution i.e. the mode of the posterior) of each individual-level TDRL parameter was determined. The resulting three parameters (i.e., learning rate, discount factor, and choice temperature) were concatenated to form a single three-vector per participant and subjected to PCA. Variables were centered and scaled prior to decomposition. Multivariate Analysis of Variance (MANOVA), using Pillai’s trace, was conducted to determine whether groups differ significantly across the multivariate space defined by the combination of all principal components (PC). Post-hoc pairwise comparisons were conducted for each PC using ANOVA with Tukey’s Honest Significance Difference test to identify specific group differences in each PC.

We performed a linear discriminant analysis (LDA) to characterize group separation in the latent parameter space captured by the PCA. We performed a LDA using each individual’s three-vector of PCs as the independent set of variables and their substance use status as the categorical grouping variable. Because individual‑level model parameters were estimated using group‑level distributions, observations were not conditionally independent thus LDA was performed without cross‑validation and results are interpreted descriptively rather than as measures of out‑of‑sample predictive performance. Group separation was summarized using the resulting discriminant function coefficients and in‑sample classification patterns derived from the confusion matrix, which are reported solely to illustrate patterns of separation in parameter space and not to assess generalization.

All analyses were conducted in R version 4.2.3.

## Results

Participant demographics and smoking histories are shown in Table 1. Two participants were excluded for not completing at least 50 trials. One participant was excluded for selecting only the easy task option. Groups did not differ in sample size (𝛘^2^(3) = 1.2, *p* = 0.753), sex distribution (𝛘^2^(3) = 5.43, *p* = 0.143), or cigarettes/day (F(2,74) = 0.40, *p* = 0.675), although they did significantly differ in age (F(3,96) = 13.88, *p* < 0.001) and racial distribution (𝛘^2^(12) = 42.80, *p* < 0.001). When comparing Shiffman-Jarvik Scores, groups did not differ in negative affect, arousal, somatic symptoms, and appetite but groups did significantly differ in craving (F(1,45) = 53.16, *p* < 0.001) and habit withdrawal (F(1,45) = 4.258, *p* = 0.0449) as expected. See Table 1 for results of group comparisons. See Supplemental Table 5 for a comparison of depression and anxiety symptoms across groups.

**Table 1:** **Participant demographics and smoking histories**. Values reported are either categorical counts (n, Sex, and Race) or mean ± standard deviation. Between-group comparisons were performed using one-way analysis of variance (ANOVA) with Bonferroni post hoc comparisons for means and chi-square tests for categorical counts. Bold *p*-values are significant

	Never TUD (*n* = 23)	Former TUD (*n* = 22)	Current TUD (*n* = 26)	TUD + OUD (*n* = 29)	Significance (*p* value)
Sex (F/M)	12 / 11	11 / 11	18 / 8	11 / 18	𝛘^2^(3) = 5.43, *p* = 0.143
Age	28.7 ± 8.9	38.5 ± 8.3	41.8 ± 10.2	30.5 ± 6.0	**F(3**,**96) = 13.88**,**p < 0.001** **Never TUD < Former TUD & Current TUD**, **TUD + OUD < Former TUD & Current TUD**
Years of education	15.7 ± 2.2	14.9. ± 2.7	13.5 ± 2.0	13.5 ± 1.5	**F(3**,**96) = 6.40**, **p < 0.001** **Never TUD > Current TUD & TUD + OUD**
Race					𝛘^2^**(12) = 42.80**, **p < 0.001**
Asian	5	1	0	0	**Asian Never TUD > all other groups**
American Indian	0	0	0	1	**Black Current TUD > TUD+OUD**
Black	8	4	16	1	**White TUD + OUD > Never TUD & Current TUD**
White	10	17	10	26	
Other	0	0	0	1	
Cigarettes/Day		17.2 ± 11.0	16.6 ± 10.3	14.9 ± 8.7	F(2,74) = 0.40, *p* = 0.675
Shiffman-Jarvik Craving		2.0 ± 1.3	5.0 ± 1.4		**F(1**,**45) = 53.16**, **p < 0.001** **Former TUD < Current TUD**
Shiffman-Jarvik Negative Affect	3.5 ± 0.9	3.8 ± 1.4	4.0 ± 1.1		F(2,68) = 1.352, *p* = 0.265
Shiffman-Jarvik Arousal	1.8 ± 1.1	2.0 ± 1.3	1.9 ± 1.1		F(2,68) = 0.004, *p* = 0.996
Shiffman-Jarvik Somatic Symptoms	5.4 ± 0.9	5.3 ± 0.9	5.4 ± 0.9		F(2,68) = 0.154, *p* = 0.858
Shiffman-Jarvik Appetite	1.4 ± 0.4	1.4 ± 0.4	1.4 ± 0.3		F(2,68) = 0.312, *p* = 0.733
Shiffman-Jarvik Habit Withdrawal		2.0 ± 1.5	2.8 ± 1.2		**F(1**,**45) = 4.258**, **p = 0.0449** **Former TUD < Current TUD**
SOWS Total Score				9.6 ± 9.9	

### EEfRT performance

Participants performed an average of 65.2 trials during the 20 min of the EEfRT (SD = 7.5, range 51–87 trials). One-way ANOVA analyses revealed groups did not differ on the number of total trials (F(3,96) = 1.191, *p* = 0.317).

Within the first 50 trials, participants made high-effort selections in 33.4% of trials, the average completion rate for high effort task selections was 79.9%, and the average completion rate for low effort task selection was 96.4%. One-way ANOVA analyses revealed groups did not differ in high effort task selection (F(3,96) = 1.094, *p* = 0.355), low effort task selection (F(3,96) = 1.094, *p* = 0.355), high effort task completion (F(3,96) = 0.491, *p* = 0.689), or low effort task completion (F(3,96) = 0.375, *p* = 0.771). High-effort selections by probability level were 12% probability level = 21.0%, 50% probability level = 33.69%, and 88% probability level = 45.65%.

### TDRL model best explains choice behavior on the EEfRT

**Fig. 2:**
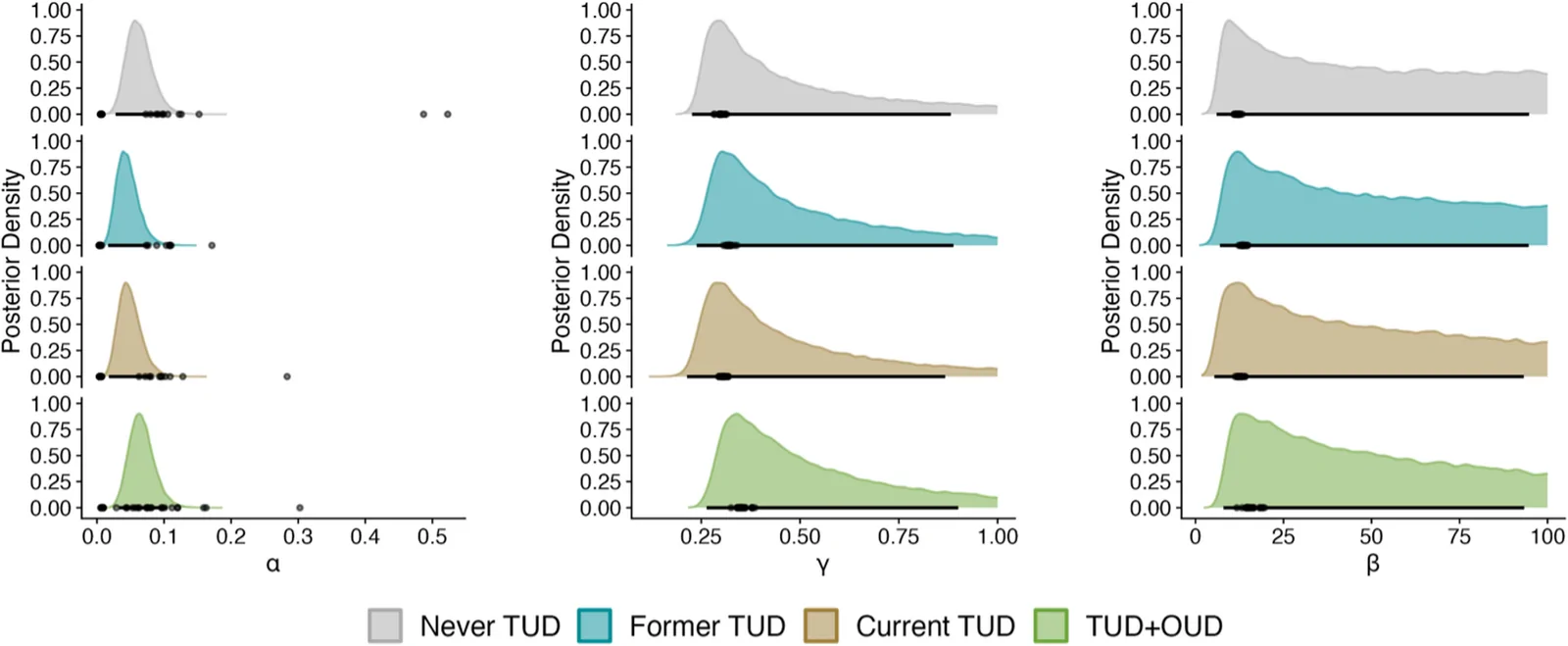
**Posterior parameter distributions for TDRL**. Each subplot displays the distributions for a distinct parameter: α – learning rate; γ – discount factor; β – inverse temperature parameter. Posterior distributions are visualized using slab densities normalized within each panel to facilitate comparison across groups. Group-level distributions are represented in the density plots. Individual-level parameter maximum a posteriori (MAP) estimated by the model are marked by points below the posterior distributions. The 95% highest density interval is indicated by the black line below the distribution

We fit the Subjective Value models and TDRL model to participant choice behavior using hierarchical Bayesian inference and compared estimates of the model evidence (i.e. marginal likelihood) and the posterior predictive accuracy (density) for all models. TDRL demonstrated greater model evidence and greater posterior predictive accuracy relative to the Subjective Value Models (Table 2), indicating that TDRL is a better description of behavior on the EEfRT and better predicts EEfRT choice behavior data. The group-level TDRL model parameters are reported in Supplemental Tables 1 and Fig. 2.

**Table 2:** **Subjective value and RL model comparison results**. Computed estimates of the Bayesian model evidence (i.e., marginal likelihood via bridge sampling) and model predictive density (i.e., expected log predictive density) for subject value and RL models. Reported values are the estimate value with interquartile range (of model evidence) and standard error reported (for predictive density) in parentheses. TDRL demonstrated the maximum (least negative) model evidence and predictive density compared to the Subjective Value models

	All Participants	
Model	Model Evidence	Model Evidence
Full SV	-3222.3 (0.03)	-3442.4 (5.40)
Reward Only	-3319.5 (0.02)	-3442.3 (5.41)
*TDRL	-2952.3 (0.72)	-3167.4 (48.88)

### Group differences in a dynamic behavioral change by substance use status

**Fig. 3:**
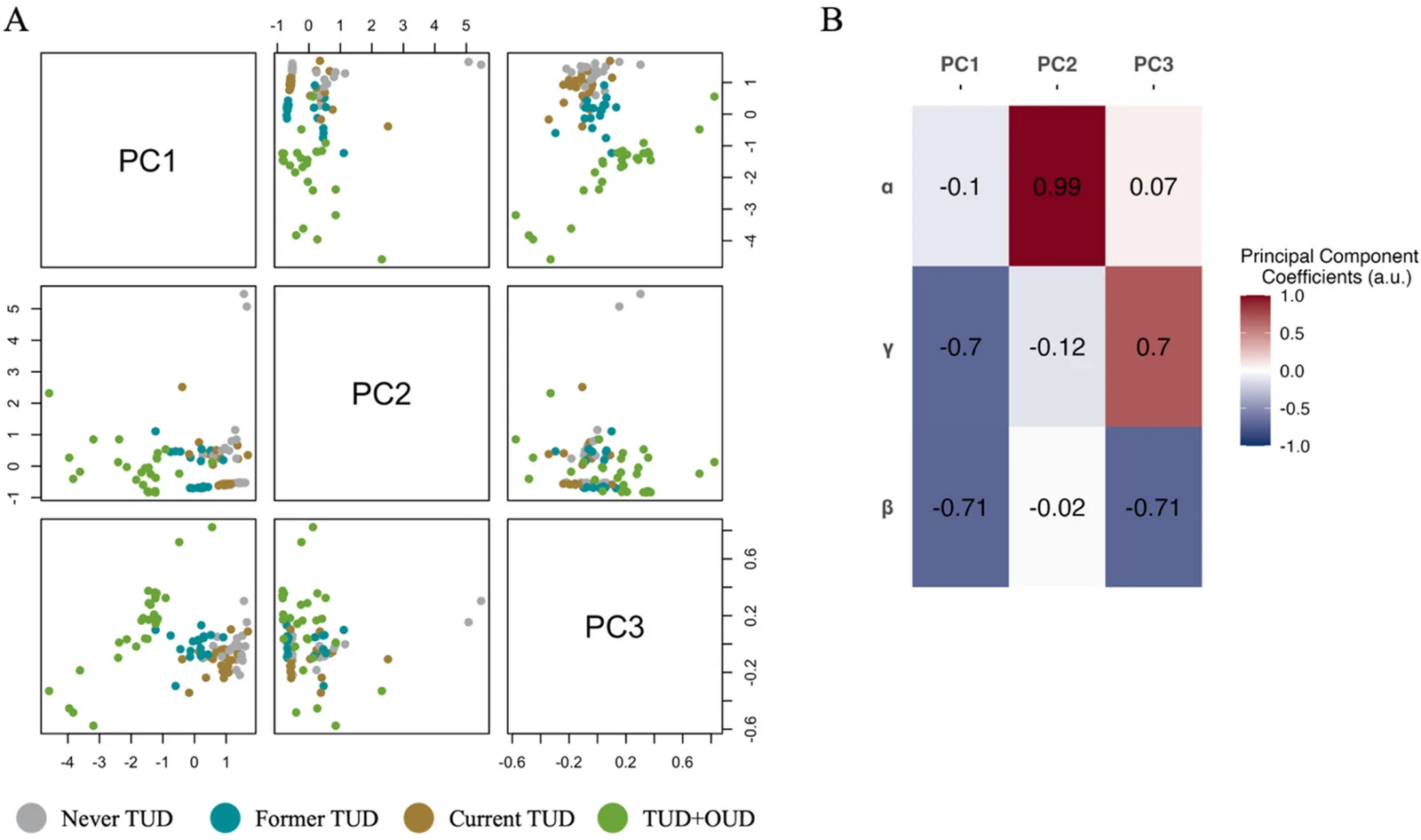
** Principal components analysis of TDRL parameters**. Principal components analysis illustrating group differences in TDRL parameters. (**A**) PCA pairs plots illustrating biplots for each and every pairing of principal components. Dots indicate individual principal component values. Colors indicate group classification of each individual. (**B**) Principal component loadings of each TDRL parameter to each principal component. Warm colors indicate a more positive loading and cool colors indicate a more negative loading

We performed a PCA to reduce dimensionality and assess whether distinct covariation amongst the three-vector of TDRL parameters could distinguish between groups. Each PC explained the following amount of the variance: PC1 = 65.4%, PC2 = 33.2%, and PC3 = 1.5%. Figure 3B details the loading strength (i.e. a coefficient that represents the strength and direction of the relationship) of each TDRL parameter for each PC. PCA revealed meaningful group differences as visualized in Fig. 3 and confirmed by MANOVA (F(3,96) = 16.08, p-value < 0.001). Post-hoc pairwise comparisons identified specific group differences in PC1 and PC3 (Supplemental Table 2). For PC1 all group pairings are significantly different excluding Never TUD compared to Current TUD. For PC2, all group pairings are not significantly different. For PC3 TUD + OUD is significantly different from all other groups.

**Fig. 4:**
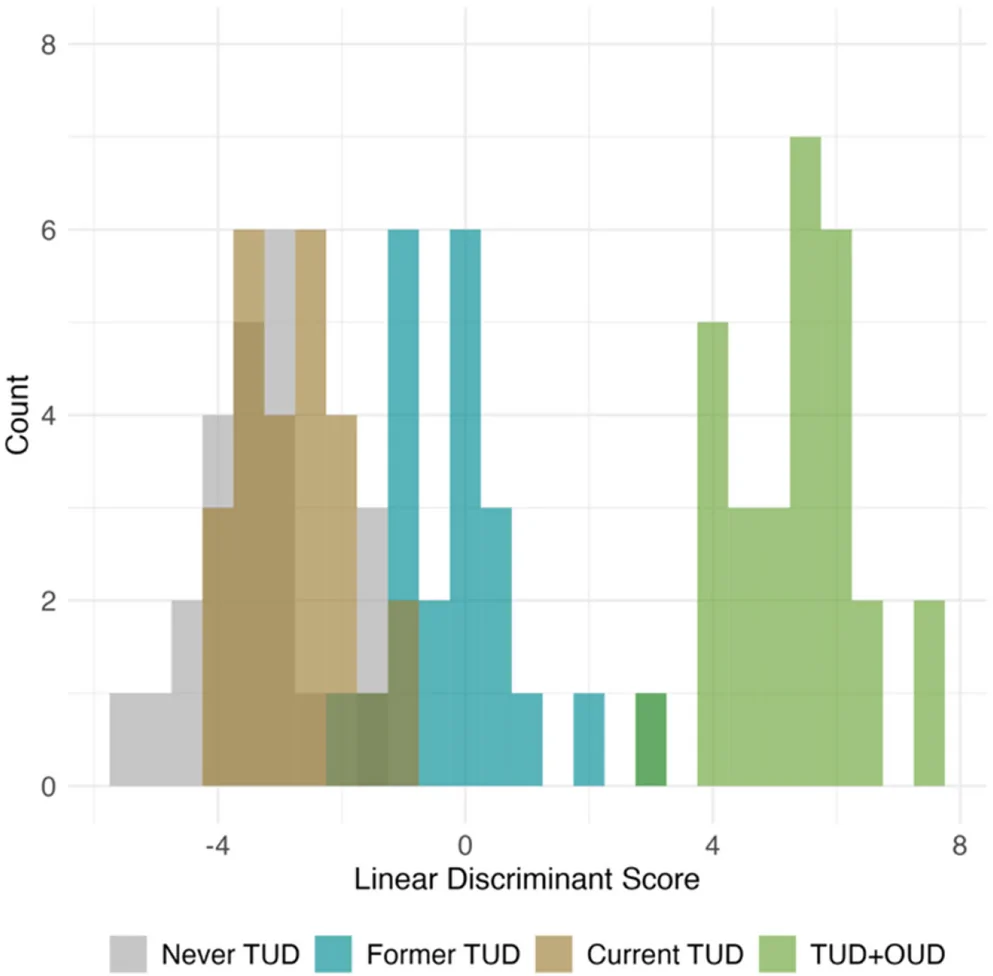
**Linear discriminant analysis of substance use**. Linear discriminant analysis illustrating group classification results based on principal component inputs. LDA histogram of the first linear discriminant dimension colored by group, illustrating group separation across discriminant dimensions. Colors indicate group classification

We performed a LDA to characterize group separation in the latent parameter space captured by the PCA, using each individual’s three-vector of PCs as the independent set of variables and their known substance use status as the categorical grouping variable. The linear discriminant coefficients (i.e. weights assigned to each PC in forming the linear discriminant axes) is as follows – LD1: PC1 = -2.40, PC2 = -0.51, PC3 = 7.36; LD2: PC1 = -0.11, PC2 = -0.82, PC3 = -2.85; LD3: PC1 = 0.29, PC2 = -0.58, PC3 = 3.31. Table 3 shows the resulting in-sample confusion matrix from the LDA. Figure 4A illustrates group separation across the first discriminant dimension. The overall classification rate is 84%, reflecting the degree of group separation within the fitted space. The LDA shows clear but non-uniform patterns of group separation in latent parameter space. The TUD + OUD group shows near-complete separation from other groups, with minimal overlap. The Former TUD group are also well separated, with most misassignments occurring with the Current TUD group. In contrast, the Never TUD and Current TUD groups exhibit the greatest overlap, with frequent misassignments.

**Table 3:** **Linear discriminant analysis confusion matrix**. In-sample confusion matrix showing patterns of group overlap and separation in parameter space from non-cross-validated LDA. Diagonal cells indicate correct group classifications. The overall classification rate is 84%

		Actual				Totals
		Never TUD	Former TUD	Current TUD	TUD + OUD	
Predicted	Never TUD	14	0	3	0	17
	Former TUD	0	20	2	0	22
	Current TUD	9	1	21	0	31
	TUD + OUD	0	1	0	29	30
	Totals	23	22	26	29	100

## Discussion

EBDM is a critical cost-benefit analysis that has been implicated in psychiatric disorders. Thus, we examined how current and former iTUD and current iOUD make effort-based decisions. As hypothesized, all groups (Never TUD, Former TUD, Current TUD, and TUD + OUD) use a RL framework to make effort-based decisions. Groups differ in their multivariate combination of TDRL posterior parameters, which accurately identifies substance use status.

Groups differ by substance use status in a dynamic behavioral change (i.e. learning) as measured by the multivariate combination of learning rate, discount factor, and choice temperature. PCA revealed potential decision-making dimensions that reliably classified groups. PC1 is the joint dimension of future reward sensitivity and choice stochasticity and it suggests (a) no difference between Never and Current TUD, (b) steeper discounting of future rewards and more stochastic/random choices characterize Former TUD when compared to Never TUD and Current TUD, and (c) steeper discounting of future rewards and more stochastic/less consistent choices characterize TUD + OUD. PC2, the learning rate dimension, showed no group differences, suggesting similar rates of updating value estimates from feedback. PC3 captures the tradeoff between future reward sensitivity and choice stochasticity and it distinguishes TUD + OUD. One potential explanation for misclassifications of Never TUD and Current TUD is current nicotine use may maintain or mask deficits in normal underlying function similar to Never TUD. Studies have shown that iTUD and controls without TUD do not differ in DA synthesis capacity (Bloomfield et al., 2014), also nicotine administration enhances other cognitive functions (i.e. spatial attention) in those with baseline deficits resulting in behaviorally normative performances (Hammersley et al., 2016). We speculate these findings could be due to Current TUD maintaining normal function through the use of nicotine. Further studies are needed to clarify these speculations, particularly with a control group with OUD only and a study design meant to study these intricacies.

EBDM has been studied in both animals and humans using paradigms that offer low-effort/low-reward options and high-effort/high-reward options (Salamone et al., 2007). The EEfRT, adapted from animal paradigms, assesses willingness to exert effort for a monetary reward (Treadway et al., 2009). Extensive research shows that DA transmission modulates effort expenditure (Assadi et al., 2009; Caul & Brindle, 2001; de Jong et al., 2015; Floresco et al., 2008; Kurniawan, 2011; Salamone et al., 2016, 2022; Wardle et al., 2011). Individuals with psychiatric disorders characterized by DA dysregulation perform differently on EBDM tasks (Barch et al., 2014; Cooper et al., 2019; Culbreth et al., 2018; Hartmann et al., 2015; Treadway et al., 2012; Treadway & Zald, 2011). SUDs have also been associated with dysregulated DA function, such as decreased endogenous DA function in chronic tobacco use (Ashok et al., 2019; Dagher et al., 2001; Fehr et al., 2008) and reduced DA transporter availability in chronic opioid use (Shi et al., 2008), which may lead to differences in EBDM performance. Prior studies have established that TDRL tracks decision-making behavior and underlying dopaminergic mechanisms (Liebenow et al., 2022; Montague et al., 1996; Sands et al., 2023a, b; Schultz et al., 1997; Sutton and Barto, 1998) but to our knowledge, no study has shown that the Subjective Value Models track underlying dopaminergic mechanisms. Consistent with this DA modulation framework, we show that TDRL better explains EBDM in healthy controls, iTUD, and iOUD.

The identified computational differences are also broadly consistent with established neuroimaging findings in TUD and OUD. Alterations in RL and value representation have been linked to dysfunctional frontostriatal circuitry including regions ventral striatum, dorsal striatum, and prefrontal regions such as the ventromedial and dorsolateral prefrontal cortex(Averbeck & O’Doherty, 2022; Kustubayeva et al., 2022; Park et al., 2017; Pratt et al., 2021), systems repeatedly implicated in both nicotine and opioid dependence. Individuals with TUD and OUD show well‑documented structural and functional abnormalities in these circuits (Klugah-Brown et al., 2020; Ma et al., 2025; McConnell et al., 2020). The present computational results provide a behavioral and algorithmic characterization of these disruptions, offering a mechanistic bridge between observable choice behavior and underlying neural alterations reported in neuroimaging studies.

TDRL, in comparison to the Subjective Value Models, offers a unifying framework for characterizing learning and choice behavioral differences that require multiple trials to observe. TDRL is defined by the ability to learn from interactions with an environment through trial and error to maximize a cumulative reward outcome. It achieves this through its ability to learn predictions of future rewards over time by updating value estimates based on differences between successive predictions. We show that this accumulation of predictive knowledge is critical for modeling EBDM, even in the absence of an explicit effort expenditure parameter. Jarvis et al. (2022) applied Rescorla-Wagner RL models to an EBDM paradigm, which combined probabilistic reward with physical effort, linking effort to learning by examining how effort modulated learning rates (Jarvis et al., 2022). Future work could extend TDRL to incorporate effort effects. Also, TDRL has been extended through Valence Partitioned Reinforcement Learning (VPRL), which partitions appetitive (Positive Valence) and aversive (Negative Valence) information into independent, parallel reward and punishment specific value systems with distinct prediction errors (Kishida & Sands, n.d.; Sands et al., 2023a, b). Future work should investigate if VPRL distinguishes reward/punishment (cost/benefit) learning better in EBDM.

This study has several limitations. First, group differences in age, years of education, and race were not controlled in the current modeling approach. However, we examined associations between TDRL posterior parameters and demographic metrics, showing significant relationships specifically for γ with demographics and β with racial distribution (see Supplement). Second, this sample made fewer high-effort selections than other community-based samples, potentially reducing generalizability (Barch et al., 2014; Huang et al., 2016). Third, one TUD + OUD participant did complete the EEfRT after buprenorphine treatment began. Fourth, an OUD without TUD participant group was not included because TUD co-morbidity is more prevalent than exclusive opioid use, as an estimated 70–85% of individuals with OUD use tobacco (John et al., 2019; McHugh et al., 2020; Parker et al., 2020). Fifth, this analysis combined two studies which brings limitations of geographical location and collection date. Sixth, the task performance monetary incentive was modest. The EEfRT incentivizes performance using a random incentive system (RIS) [see Treadway et al. (2009)], where one trial is randomly selected for payment, and this method is commonly used in behavioral economic experiments (Lee, 2008; Starmer & Sugden, 1991). Empirical evidence shows that behavior under RIS closely tracks behavior under full-payment schemes, supporting its validity as an incentive mechanism (Koppel et al., 2026) and recent work has shown that the size of the reward does not have an effect on performance or self-reported effort and intrinsic motivation (Holst-Hansen & Bergenholtz, 2020). Seventh, while this work demonstrated alterations in EBDM in individuals with current and past TUD, this cross-sectional design cannot help determine if these differences are a cause or consequence of cessation. A longitudinal study is needed to investigate whether these decision-making alterations recover with cessation. Additionally, SUDs are frequently co-morbid with depression and anxiety symptoms, which may also influence EBDM. Some of the participants reported experiencing these symptoms; however, this study was not powered to investigate the influence of these symptoms on EBDM. Future work should disentangle the potential impact of comorbid psychiatric conditions to understand how comorbidities can influence EBDM and learning. Computational approaches may provide the granularity needed to sort out these complex cases (Huys et al., 2016; P. R. Montague et al., 2012; Wang & Krystal, 2014). Lastly, while results are consistent with dopaminergic accounts of effort, this interpretation remains speculative and direct investigation is needed.

Cigarette smoking is the leading preventable cause of disease, death, and disability in the United States, accounting for approximately 16 million smoking-related diseases and 480,000 deaths annually (Centers for Disease Control and Prevention, 2024). In 2023, nearly 80,000 people died from an opioid-involved overdose, making up about 76% of all drug overdoses (Centers for Disease Control and Prevention, 2025). Also, over 80% of individuals with opioid use disorder use tobacco (John et al., 2019). The observed group differences in computational parameters governing effort-based decision‑making suggest that substance use and comorbidity are associated with systematic alterations in how individuals integrate feedback, update expectations, and guide future choices. Such processes are directly relevant to treatment engagement in SUD, where patients must repeatedly learn from delayed, probabilistic outcomes and weigh rewards to effort expenditure. From a translational perspective, computational markers of learning and valuation may help explain why individuals with greater substance use severity or comorbidity show poorer adherence to treatment and elevated relapse risk, even when motivation is present. These parameters may therefore represent cognitive targets for intervention by identifying individuals who may benefit from strategies that reduce decision noise, enhance sensitivity to negative feedback, or scaffold learning in early treatment phases.

## Supplementary Information

Below is the link to the electronic supplementary material.

Supplementary Material 1 (DOCX 1.88 MB)

## Data Availability

The data that support the findings of this study are available from the corresponding author upon reasonable request. Custom-written analysis scripts for generating the behavioral results of this manuscript are available on GitHub (https://github.com/KaseyPSpry/Article-Effort_Based_Decision_Making_in_Tobacco_Use.git).
