# Supplementary material for: Effort and substance use: differentiating tobacco use through reinforcement learning of effort based decision making
Source: J Comput Neurosci. 2026 Aug 28;54(4):709–23. doi: 10.1007/s10827-026-00953-6 (PMC13710678; doi:10.1007/s10827-026-00953-6)
Supplement: Supplementary file 1 [file 10827_2026_953_MOESM1_ESM.docx]

**Effort and Substance Use: Differentiating Tobacco Use Through Reinforcement Learning of Effort Based Decision Making**

**Authors:** Kasey P. Spry^1,2^, Jazmyne James^1^, Alison H. Oliveto^3^, Michael Mancino^3^, Kenneth T. Kishida^4,1,5 †^ , Merideth A. Addicott^1†^

**Affiliations:**

^1^ Department of Translational Neuroscience, Wake Forest University School of Medicine, Winston-Salem, NC, USA

^2^ Neuroscience Graduate Program, Wake Forest University Graduate School of Arts and Sciences, Winston-Salem, NC, USA

^3^ Department of Psychiatry , University of Arkansas for Medical Sciences, Little Rock, AR, USA

^4^ Department of Biology, Wake Forest University, Winston-Salem, NC, USA

^5^ Department of Neurosurgery, Wake Forest University School of Medicine, Winston-Salem, NC, USA

^†^ Merideth A. Addicott and Kenneth T. Kishida are co-senior authors

**Corresponding Authors:**

Merideth A. Addicott: [Merideth.Addicott@wfusm.edu](mailto:Merideth.Addicott@wfusm.edu)

Kenneth T. Kishida: [kishidk@wfu.edu](mailto:kishidk@wfu.edu)

**SUPPLEMENTAL METHODS, RESULTS AND FIGURES**

**Power Analysis**

Since we compared all pairwise combinations of groups for a total of 6 comparisons we used a Bonferroni correction for multiple comparisons, so each comparison is made at the 0.008 alpha level to preserve an overall type I error of 0.05 across all comparisons. We present power calculations for comparing the 2 groups with the smallest sample sizes, Never TUD and Former TUD, as other comparisons would achieve at the least the power presented here. We used a two-sample two-sided t-test on β to compare groups. We can detect a difference of 1.13 standard deviations with 80% power.

**TDRL Group Comparison in Univariate Parameter Results**

Given that participant choice behavior is better explained by TDRL, we investigated group differences in the posterior parameter distributions. The group-level TDRL model parameters are reported in Supplemental Table 1 and Figure 2. Supplemental Figure 1 reports posterior distribution of differences between each and every group. Evidence of group distributional differences is present when the 95% highest density interval (HDI) excluded 0 or was within 0.05 of excluding 0 (Kruschke, 2010). At the group-level there were no credible differences between groups for individual α, γ, and β parameters (Supplemental Figure 1).

**Participant Demographics and TDRL Posterior Parameters**

We investigated if there were relationships between demographics and TDRL posterior parameters as groups differed in three demographic categories (age (F(3,96) = 13.88, p < 0.001), years of education (F(3,96) = 6.40, p < 0.001), and racial distribution (𝛘^2^(12) = 42.80, p < 0.001); Table 1). Individual-level TDRL parameter estimates, maximum a posteriori estimate (MAP; the parameter value that maximizes the posterior distribution i.e. the mode of the posterior) were used as a summary statistic for each participant. Pearson correlations were conducted to assess the relationship of TDRL parameters with continuous variables and one-way analysis of variance (ANOVA) to assess the relationship of TDRL parameters with categorical variables. When assessing race, participants that identified as American Indian, Asian, Black, and other were grouped together in an overall Minority group due to the limited numbers of some racial categories.

Learnrate (α) did not have a significant relationship with age (r(98) = -0.127, p-value = 0.209), years of education (r(98) = 0.021, p-value = 0.838), nor racial distribution (F(1,98) = 0.098, p-value = 0.755). Discount factor (γ) did have a significant relationship with age (r(98) = -0.204, p-value = 0.042; Supplemental Fig. 2A), years of education (r(98) = -0.197, p-value = 0.050; Supplemental Fig. 2B), and racial distribution (F(1,98) = 19.92, p-value < 0.001; Supplemental Fig. 2C). Inverse choice temperature (β) did have a significant relationship with racial distribution (F(1,98) = 15.05, p-value < 0.001; Supplemental Fig. 2D) and but did not have a significant relationship with age (r(98) = -0.190, p-value = 0.058) nor years of education (r(98) = -0.193, p-value = 0.054).

**Linear Discriminant Analysis Sensitivity Analysis**

To assess whether group separation in multivariate parameter space was driven by demographic differences or parent study, we conducted a series of sensitivity analyses. Sensitivity analyses were conducted in three ways. First, demographic variables were included directly as covariates in the LDA to evaluate whether group separation persisted after accounting for demographic differences. Second, each principal component was residualized with respect to age, years of education, and race, and the LDA was repeated using the residualized components to isolate demographic‑independent structure. Third, to provide a complementary and interpretable assessment, scores on the first linear discriminant (LD1) were regressed on group while adjusting for age, years of education, and race.

The original LDA revealed strong multivariate separation between groups, dominated by the first discriminant function (LD1 singular value = 20.69). Including age, years of education, and race as covariates in the LDA did not reduce discriminant separation (LD1 singular value = 21.33), indicating that demographic variables did not account for the primary axis of group differentiation. Residualizing principal components with respect to age, education, and race attenuated but did not eliminate discriminant separation (LD1 singular value = 6.90), suggesting that demographic factors explain some shared variance while leaving substantial group‑related structure intact. Consistent with these findings, regression of LD1 on group while adjusting for age, years of education, and dichotomized race (White vs. minority) showed that group remained a strong predictor of LD1 (Never TUD: β = −3.42, p-value < 0.001; Current TUD: β = −2.45, p-value < 0.001; TUD+OUD: β = 5.41, p-value < 0.001), whereas demographic variables were not independently associated with LD1 (age: p-value = 0.348; years of education: p-value = 0.324). Together, these findings indicate that the primary axis of multivariate separation reflects group‑related differences beyond demographic characteristics.

**Model Recovery and Parameter Recovery**

To validate that the models considered in this study (TDRL, Full SV, and Reward Only SV models) could be reliably distinguished and that our model comparison analysis is able to accurately identify the true generative model of choice behavior on the EEfRT, we performed a model recovery analysis. For this model recovery analysis, we simulated choice behavior on the EEfRT for all participants using the MAP individual-level parameter values for TDRL, Full SV, and Reward Only SV models and then computed model comparison criteria to determine whether the model comparison analysis identified the true generative model. Across all three generative models, the recovery results showed a consistent pattern: TDRL was the best‑fitting model regardless of the model used to generate the data (Supplemental Table 3). This pattern indicates that TDRL can approximate the behavioral patterns produced by both simpler models, whereas the simpler models cannot approximate the dynamics captured by TDRL. Rather than indicating a flaw in TDRL this pattern reflects the relationship between these models: TDRL is a more flexible, more general, and biologically grounded as seen in the literature. This finding aligns with the literature showing extensive behavioral and neural evidence that temporal difference learning is the mechanism by which reward prediction and decision making occur.

This model recovery analysis also suggests that Full SV and Reward Only SV models are useful as descriptive models, but they might not fully capture the cognitive and neural mechanisms of decision making in the EEfRT whereas TDRL provides a mechanistic account of decision making captured in the EEfRT. Altogether this model recovery analysis shows that even if participants choice behavior resembles these descriptive models, this choice behavior is still best understood as arising from the underlying temporal-difference learning mechanisms rather than from fundamentally distinct computational mechanisms.

To validate that our model fitting procedure is able to accurately estimate model parameters for each participant, we performed a parameter recovery analysis. We first calculated the MAP for each parameter for each participant to simulate choice datasets (n = 100) on the EEfRT. The TDRL model was refitted to the simulated datasets and then we computed the Pearson’s correlation coefficient between the MAP of the model parameters fitted to the actual participant data and the simulated data. All model parameters are recoverable with statistically significant moderate to strong correlations (Supplemental Table 4).

**Comparison of Depression and Anxiety Between Groups**

Evidence suggests that depression is characterized by impairments in motivation and effort allocation, often referred to as motivational anhedonia, and are less willing to exert effort for rewards on laboratory paradigms such as the EEfRT (Treadway et al., 2012; Treadway & Zald, 2011). Although less extensively studied, anxiety has also been linked to altered cost-benefit evaluation and decision-making processes, particularly in contexts involving uncertainty and potential threat (Hartley & Phelps, 2012). Participants in these studies were screened for depression and anxiety symptoms.

In study 1, a subsample of participants (n = 43) completed the Center for Epidemiologic Studies Depression Scale (CES-D; Radloff, 1977) and depression severity categories were derived using established CES-D cutoffs. In study 2, participants completed the Hamilton Depression Rating Scale (HAM-D; Hamilton, 1960) and depression severity categories were derived using established HAM-D cutoffs. Depression severity was categorized as none, mild, moderate, or severe. A chi-square test compared the distribution of severity categories across groups, revealing a significant association between group and depression severity category (Supplemental Table 5; 𝛘^2^(9) = 30.10, p < .001). Examination of standardized residuals indicated that participants in the TUD+OUD group were overrepresented in the mild (standardized residual = 3.53) and moderate (standardized residual = 2.82) depression categories and underrepresented in the no-symptoms category (standardized residual = −3.99). In contrast, Never TUD were overrepresented in the no-symptoms category (standardized residual = 2.36) and underrepresented in the mild depression category (standardized residual = −2.23). Former TUD were overrepresented in the severe depression category (standardized residual = 2.11) and underrepresented in the mild depression category (standardized residual = −2.14).

In study 1, a subsample of participants (n = 43) completed the Beck Anxiety Inventory (BAI; (Beck et al., 1988) and anxiety severity categories were derived using established BAI cutoffs. In study 2, participants completed the Hamilton Anxiety Rating Scale (HAM-A; Hamilton, 1959) and anxiety severity categories were derived using established HAM-A cutoffs. Anxiety severity was categorized as none, mild, moderate, or severe. A chi-square test compared the distribution of severity categories across groups, revealing a non-significant association between group and anxiety severity category (Supplemental Table 5; 𝛘^2^(9) = 3.86, p = 0.920).

| **Supplemental Table 5. Comparison of Depression and Anxiety Between Groups.** Values reported are categorical counts. Between-group comparisons were performed using chi-square tests for categorical counts. Bold p-values are significant. | | | | | |  |
| --- | --- | --- | --- | --- | --- | --- |
|  | Never TUD  (n = 15) | Former TUD  (n = 14) | Current TUD  (n = 14) | TUD+OUD  (n = 29) | Significance  (p value) | |
| Depression Symptoms |  |  |  |  | **𝛘^2^(9) = 30.10, p < 0.001** | |
| None | 14 | 12 | 11 | 12 |  | |
| Mild | 0 | 0 | 3 | 12 |  | |
| Moderate | 0 | 0 | 0 | 5 |  | |
| Severe | 1 | 2 | 0 | 0 |  | |
| Anxiety Symptoms |  |  |  |  | 𝛘^2^(9) = 3.86, p = 0.920 | |
| None | 10 | 6 | 7 | 26 |  | |
| Mild | 4 | 5 | 5 | 11 |  | |
| Moderate | 1 | 2 | 1 | 1 |  | |
| Severe | 0 | 1 | 1 | 2 |  | |
